# Molecular dynamics simulations support a multistep pathway for activation of branched actin filament nucleation by Arp2/3 complex

**DOI:** 10.1016/j.jbc.2023.105169

**Published:** 2023-08-16

**Authors:** Yuvraj Singh, Glen M. Hocky, Brad J. Nolen

**Affiliations:** 1 Department of Chemistry, New York University; 2 Simons Center for Computational Physical Chemistry, New York University; 3 Department of Chemistry and Biochemistry, Institute of Molecular Biology, University of Oregon

**Keywords:** Arp2/3 complex, actin, WASP, molecular dynamics, nucleation

## Abstract

Actin-related protein 2/3 complex (Arp2/3 complex) catalyzes the nucleation of branched actin filaments that push against membranes in processes like cellular motility and endocytosis. During activation by WASP proteins, the complex must bind WASP and engage the side of a pre-existing (mother) filament before a branched filament is nucleated. Recent high-resolution structures of activated Arp2/3 complex revealed two major sets of activating conformational changes. How these activating conformational changes are triggered by interactions of Arp2/3 complex with actin filaments and WASP remains unclear. Here we use a recent high-resolution structure of Arp2/3 complex at a branch junction to design all-atom molecular dynamics simulations that elucidate the pathway between the active and inactive states. We ran a total of ∼4.6 microseconds of both unbiased and steered all-atom molecular dynamics simulations starting from three different binding states, including Arp2/3 complex within a branch junction, bound only to a mother filament, and alone in solution. These simulations indicate that the contacts with the mother filament are mostly insensitive to the massive rigid body motion that moves Arp2 and Arp3 into a short pitch helical (filament-like) arrangement, suggesting actin filaments alone do not stimulate the short pitch conformational change. In contrast, contacts with the mother filament stabilize subunit flattening in Arp3, an intrasubunit change that converts Arp3 from a conformation that mimics an actin monomer to one that mimics a filamentous actin subunit. Our results support a multistep activation pathway that has important implications for understanding how WASP-mediated activation allows Arp2/3 complex to assemble force-producing actin networks.

Filament nucleation is a critical step in the regulation of the actin cytoskeleton, as it controls when and where actin filament networks assemble in cells (1). Spontaneous nucleation of actin filaments is energetically unfavorable, but once nucleated, new actin monomers can elongate both the fast-growing barbed end and the slow-growing pointed end of filaments (2). Cells rely on multiple classes of actin filament nucleators to catalyze nucleation and direct the assembly of new actin filaments, including tandem WH2 domain-containing proteins, formins, and actin-related protein 2/3 complex (Arp2/3 complex) (1, 3). Among these nucleators, Arp2/3 complex is the only one that can nucleate branched actin filaments (4). Branched actin networks assembled by Arp2/3 complex play important roles in endocytosis, cellular migration, maintenance of cell–cell junctions, meiosis, DNA repair, and vesicle trafficking (5, 6).

To properly orchestrate complex cellular functions, the activity of Arp2/3 complex must be regulated so that nucleation occurs at the right time and location within the cell. On its own, the complex is inactive. Activation requires binding to a nucleation-promoting factor protein (1, 7). WASP family proteins form a class of nucleation-promoting factors that activate Arp2/3 complex to create branches, but WASP is insufficient for activation; activation also requires that WASP recruit actin monomers to the complex and that the complex bind to a pre-existing “mother” filament of actin (7, 8, 9). Upon activation by WASP, Arp2/3 complex nucleates a new actin filament with a free barbed end and its pointed end anchored to the complex at the newly formed branched actin filament junction (10). How the nucleation activity of Arp2/3 complex is triggered by binding of WASP, WASP-recruited actin monomers, and actin filaments is unclear, despite its implications for understanding a wide range of cellular processes.

High-resolution structures of Arp2/3 complex in the inactive state have been available since 2001 (11), but high-resolution structures of the activated complex have only recently become available because of advances in cryo-electron microscopy (cryo-EM) methods (12). Among the recently solved cryo-EM structures are (a) a 9.0 Å structure of activated human Arp2/3 complex reconstructed from branch junctions imaged in cells (13), (b) a 3.9 Å structure of activated *Bos taurus* Arp2/3 complex at a branch junction (14), and (c) 3.5 Å and 3.9 Å structures of activated *Schizosaccharomyces pombe* Arp2/3 complex (15, 16). These structures, along with biochemical data (17, 18), confirmed the long-standing hypothesis that during nucleation, the two actin-related subunits in the complex—Arp2 and Arp3—mimic a filamentous actin dimer to template the growth of a new filament (11). Along with the previously solved inactive structures, the new structures revealed two major structural changes that bring Arp2 and Arp3 into a filamentous dimer-like conformation (14, 15, 16). First, twisting of clamp subunits ARPC2 and ARPC4 rotates the bottom half of the complex (subunits ARPC1, ARPC5, Arp2, and the globular portion of ARPC2) to move Arp2 and Arp3 from an end-to-end (“splayed”) conformation into an arrangement that mimics the positioning of two consecutive actin subunits along the short pitch helical axis of a filament (Video S1). Second, Arp2 and Arp3 each transition from a twisted state to a flattened state, an intramolecular change in which the four subdomains of each Arp move into approximately the same plane (Video S1). Flattening also occurs in actin when it transforms from a monomeric to a filamentous state (19, 20, 21), indicating this change is a key step in allowing the Arps to mimic a filamentous actin dimer. Flattening of the Arps is thought to trigger opening of grooves on their barbed ends for interactions with the first actin monomers in the newly nucleated (daughter) filament (14, 15, 16).

While the new structures revealed the key conformational changes required for activation, how binding of WASP, WASP-recruited actin monomers, and actin filaments stimulates these structural changes remains unclear. One model postulates that the structural changes are concerted (or strongly coupled) and that the activating factors bind cooperatively to Arp2/3 complex to stimulate them in a single step (Figs. 1*A* and S1) (15). Two main observations support the concerted model. First, under some conditions, actin filaments increase the binding affinity of WASP for Arp2/3 complex, indicating cooperativity between WASP and filaments. Second, a large surface area is buried when Arp2/3 complex binds actin filaments, suggesting the availability of large amount of binding energy to stimulate major conformational changes (9, 15, 22, 23). A second model proposes that activation occurs *via* multiple steps (Fig. 1*A* and S1). In one step, WASP and WASP-recruited actin monomers stimulate movement of the complex into the short pitch conformation. In another step, bound actin filaments stimulate subunit flattening. The multistep model is supported by engineered crosslinking assays that show WASP and WASP-recruited actin monomers stimulate movement into the short pitch conformation (17, 24), but actin filaments do not (24). In addition, structures of activated Arp2/3 complex at a branch junction revealed interactions with actin filaments that can be made in the flattened but not the twisted conformation of Arp3, suggesting actin filaments stimulate subunit flattening (14). Distinguishing between activation mechanisms is critical for understanding how the complex serves as a “coincidence detector” that triggers nucleation only when WASP, WASP-recruited actin monomers, and actin filaments are bound. The stringent requirement for each activator is thought to be critical for the ability of Arp2/3 complex to assemble functional actin networks in cells. For instance, the requirement for actin filaments ensures that when activated by WASP, Arp2/3 complex nucleates only branched actin filaments, which are optimal for pushing against broad flat surfaces like the plasma membrane at the leading edge of lamellipodia within motile cells (25, 26). The requirement for WASP-recruited actin monomers helps control the density of branches nucleated within Arp2/3 complex assembled actin networks (27, 28). The requirement for WASP connects Arp2/3 complex to cellular signaling pathways and targets the branching nucleation activity of Arp2/3 complex to the proper cellular location (5, 29). Therefore, understanding how each of these factors contributes to the activating conformational changes in Arp2/3 complex is critical for understanding how the complex assembles force-producing actin networks in cells.

Computational simulations provide a powerful tool to understand the dynamics of biomolecular structures (30). In the case of Arp2/3 complex, previous simulations have yielded important insights into multiple aspects of activation and nucleation. For instance, atomistic and coarse-grained molecular dynamics (MD) simulations have uncovered details about the binding of WASP and actin monomers to the complex and changes in its conformation caused by nucleotide binding and hydrolysis (31, 32, 33). Unbiased all-atom MD simulations investigated the role of adenine nucleotides in controlling the conformation of Arp3 and Arp2, the branch angle and stability, and details of the interface of the complex with the mother filament (31, 34, 35). All-atom steered molecular dynamics (SMD) showed that movement of Arp2/3 complex into the short pitch conformation occurs *via* twisting of the clamp subunits, ARPC2 and ARPC4 (36). While each of these approaches has its strengths and weaknesses, all-atom MD simulations can be particularly useful because they provide atomistic details of the structural rearrangements that occur when a macromolecule transitions between states. However, such simulations are more informative if high-resolution structures are available to define each of the endpoints of a conformational pathway, thereby tethering simulation trajectories to the empirical data at multiple points along the pathway (37). Therefore, the recent availability of high-resolution structures of activated Arp2/3 complex marked an important increase in the potential of all-atom simulations to yield insights into the pathway to nucleation by Arp2/3 complex.

Here we take advantage of the recently solved structure of activated *B. taurus* Arp2/3 complex bound at a branch junction—along with a high-resolution X-ray crystal structure of the inactive *B. taurus* Arp2/3 complex—to investigate the pathway between active and inactive states of Arp2/3 complex (14, 38). We ran microsecond unbiased MD simulations starting from the active conformation of Arp2/3 complex anchored at a branch junction, bound to the side of an actin filament, or free in solution, along with a microsecond simulation of the complex free in solution starting from an inactive state. We also used SMD to pull Arp2/3 complex from the active to an inactive state when it was bound to the side of a filament. These data support a multistep activation pathway of Arp2/3 complex in which WASP and actin monomers stimulate the short pitch conformation and actin filaments stimulate subunit flattening. Specifically, the simulations provide evidence that actin filaments trigger only one of the two conformational changes required for activation: subunit flattening. They also reveal an intermediate conformation in which the complex is in the short pitch conformation, but Arp2 and Arp3 are twisted into the monomer-like conformation, a result that argues against a concerted mechanism. By providing information that allows us to distinguish between activation mechanisms, these simulations provide important insights into the conformational pathway to Arp2/3 complex activation by WASP.

## Results

### Description of system setup

**Figure 1:**
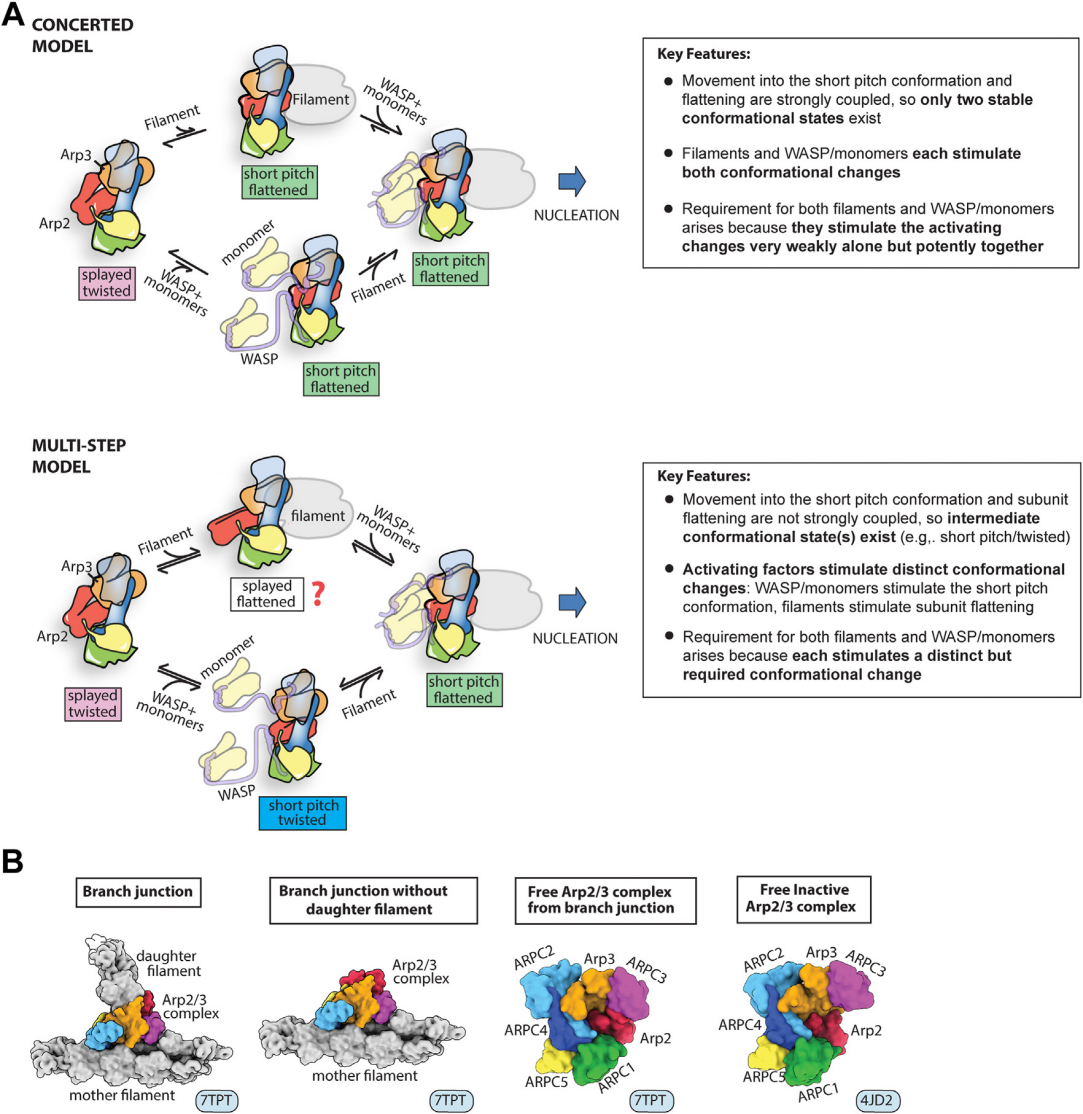
**Proposed mechanisms for pathway to Arp2/3 complex activation and overview of simulation setup.***A*, simplified schematics of the conformational pathway to activation of Arp2/3 complex in a concerted *versus* multistep model of activation. Previous data indicate that while WASP triggers the splayed to short pitch conformational change, both states can exist with or without WASP bound to the complex (17, 18). For clarity, neither these conformational states nor their reversibility is depicted here. For a more detailed diagram of the two proposed mechanisms that includes these states, see Fig. S1. Text boxes to the right of each scheme list the key features of each mechanism. The splayed/flattened state (marked with *red question mark*) may not be adopted because of steric clash (see Discussion). *B*, starting structures used for each of the four unbiased all-atom MD simulations described here. The PDB file used to build each structure is indicated in the lower right corner. Arp2/3 complex, actin-related protein 2/3 complex; MD, molecular dynamics.

Four different systems were set up for MD simulations (Fig. 1*B*). The branch junction simulation was built from the recent cryo-EM structure of activated *B. taurus* Arp2/3 complex at a branch junction (PDB ID 7TPT, (14)). In this simulation, Arp2/3 complex was bound to the side of a mother filament consisting of 10 actin subunits and to the pointed end of the nucleated daughter filament containing four actin subunits, as in the cryo-EM reconstruction (14). The branch junction without daughter filament simulation is identical to the branch junction simulation, except that the daughter filament was removed. In the simulation of free Arp2/3 complex from the branch junction, both the mother filament and daughter filament were removed from 7TPT. These three simulations allowed us to take advantage of the newly solved structures of Arp2/3 complex in the activated state, but it is important to note that any movement of these structures toward the inactive state represents the reverse direction of conformational changes that would be observed during the activation process. Lastly, the free inactive Arp2/3 complex simulation was built from the crystal structure of inactive *B. taurus* Arp2/3 complex bound to the inhibitor GMFγ (PDB ID 4JD2), as this structure provides the most complete model of inactive Arp2/3 complex (38). GMFγ was removed from the coordinate file for the free inactive Arp2/3 complex simulation. In all but the branch junction simulation, Arp2 and Arp3 had bound ATP in their nucleotide clefts, consistent with a preactivation state. In the simulation of the branch junction, we modeled ADP into the clefts of Arp2 and Arp3, because both Arp2 and Arp3 hydrolyze ATP after branch formation (13, 39, 40, 41). Similarly, ATP is hydrolyzed by actin subunits upon polymerization (42), so ADP is present in the nucleotide clefts of mother and daughter actin in the cryo-EM reconstruction and in the simulations here (14). All systems were set up using the CHARMM22+CMAP forcefield with explicit TIP3P water and 50 mM neutral salt concentration, using K^+^ and Cl^-^ ions (43). Further details on system construction, minimization, heating, and production can be found in the Experimental Procedures. System sizes ranged from four hundred thousand to 1.4 million atoms. Due to the relatively large size of these systems, we limited the simulation times to 1 μs, a duration that—as described below—may not be long enough to reach the most stable ground state conformations for some of the systems.

The SMD simulations were run exclusively on the branch junction without daughter filament, with the goal of pulling Arp2/3 complex from the short pitch to the splayed conformation while it remained bound to the side of a filament. A harmonic biasing restraint was applied by defining the center of geometry (COG) of each of the subunits and applying a spring constant of 10,000 kJ•mol^−1^•Å^−2^ between each COG. The relaxed spring length for each subunit pair was the distance between the centers of mass of each subunit when the complex was in the splayed conformation (as defined by the structure of the inactive Arp2/3 complex, 4JD2). We ran three different simulations in which the bias was applied over 60, 100, and 150 ns, after which the simulation was continued without the restraint to a total simulation time of ∼200 ns.

### The splayed and short pitch conformations are maintained in microsecond unbiased MD simulations

**Figure 2:**
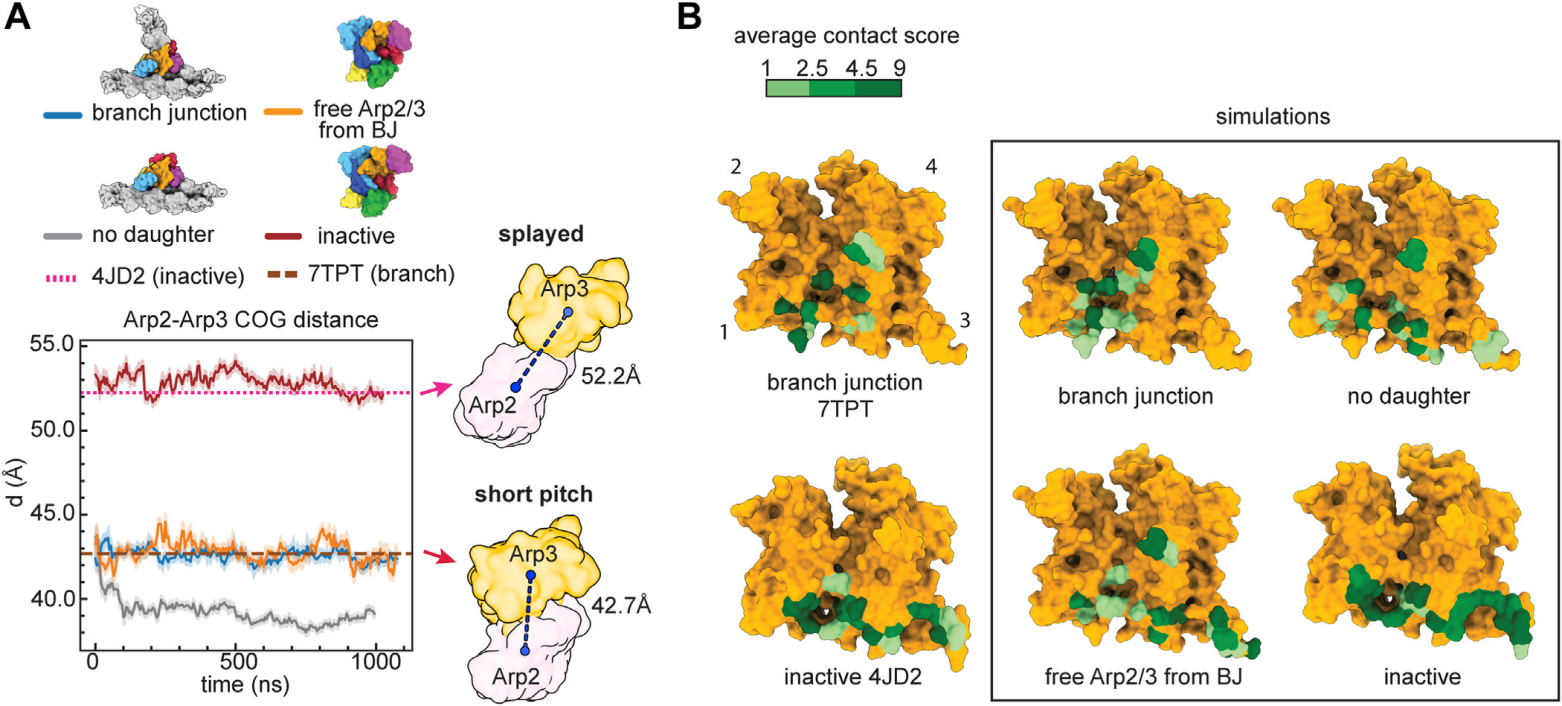
**The splayed and short pitch conformations are maintained in microsecond unbiased MD simulations**. *A*, plot of the distance of the center of geometry (COG) of subdomains 3 and 4 of Arp2 to the COG of subdomain 3 and 4 of Arp3 as a function of simulation time. *Dashed* and *dotted lines* show the corresponding distance in the branch junction structure, 7TPT, and the inactive Arp2/3 complex structure, 4JD2, respectively. Data for simulations are shown as average smoothed over 5 ns (50 frames) for this and all other plots from the unbiased simulations. The *shaded area* shows the standard deviation over the smoothing window. *B*, surface representation of Arp3 from inactive (4JD2) or active structure (7TPT) showing average contact scores over the entire trajectory for Arp3 residues that contact Arp2. Contact scores for 4JD2 and 7TPT are shown on the left for reference. Contact scores were calculated using PyContact, as described in the methods. Arp2/3 complex, actin-related protein 2/3 complex; MD, molecular dynamics.

We first asked whether Arp2/3 complex switched between the splayed and short pitch conformations in each of the simulations. We tracked this conformational change by measuring the distances between the COG of the inner domain (subdomains 3 and 4) of Arp2 with the inner domain (subdomains 3 and 4) of Arp3. High-resolution structures show that this distance decreases from 52.2 to 42.7 Å during the short pitch conformational switch (14, 38). In the simulation of free inactive Arp2/3 complex, Arp3 and Arp2 remain splayed, with residues that stabilize the splayed interface remaining in close contact throughout the simulation (Fig. 2, *A* and *B*). This result is consistent with previous biochemical and structural data showing that splayed conformation is strongly favored in the absence of activating factors (17, 18, 44). In contrast, analysis of cryo-EM structures showed that the short pitch conformation is stabilized in the context of the branch junction (14, 15). Consistent with these data, the short pitch conformation is maintained throughout the entire 1 μs branch junction simulation, with an average distance of 42.6 Å, very close to the distance in the branch junction cryo-EM structure (Fig. 2, *A* and *B*). Despite the loss of stabilizing interactions, the short pitch conformation was also maintained over the entire microsecond trajectory in the simulations of the branch junction without daughter filament and the free Arp2/3 complex from the branch junction (Fig. 2*A*). In both simulations, the Arp2 and Arp3 maintained contacts characteristic of the short pitch arrangement (Fig. 2*B*). Assuming the simulations represent a near average behavior, they point to an energy barrier of at least several *k*_*B*_T between the splayed and short pitch conformations (45). The stability of the short pitch state over the entire 1 μs simulation is consistent with experimental and theoretical studies that estimate rigid body motions in proteins to be on the order of micro-to milliseconds (45), although we would expect that the complex would relax to the splayed conformation in longer simulations of the branch junction or the branch junction without the daughter filament.

During the simulation of the branch without daughter filament, the COG of the inner domains of Arp2 and Arp3 moved together by ∼4.0 Å (Fig. 2*A*). This is a result of Arp2 tilting toward the barbed end of Arp3, making additional contacts to Arp3, but adopting a conformation that would clash with actin subunit DA1 from the daughter filament (Fig. S2). Therefore, this state, which is stable throughout the trajectory, would not be expected to be nucleation competent, even though it has a conformation close to the short pitch arrangement seen in the branch junction structure (Fig. S2).

### Flattening and adoption of the short pitch conformation are not tightly linked

**Figure 3:**
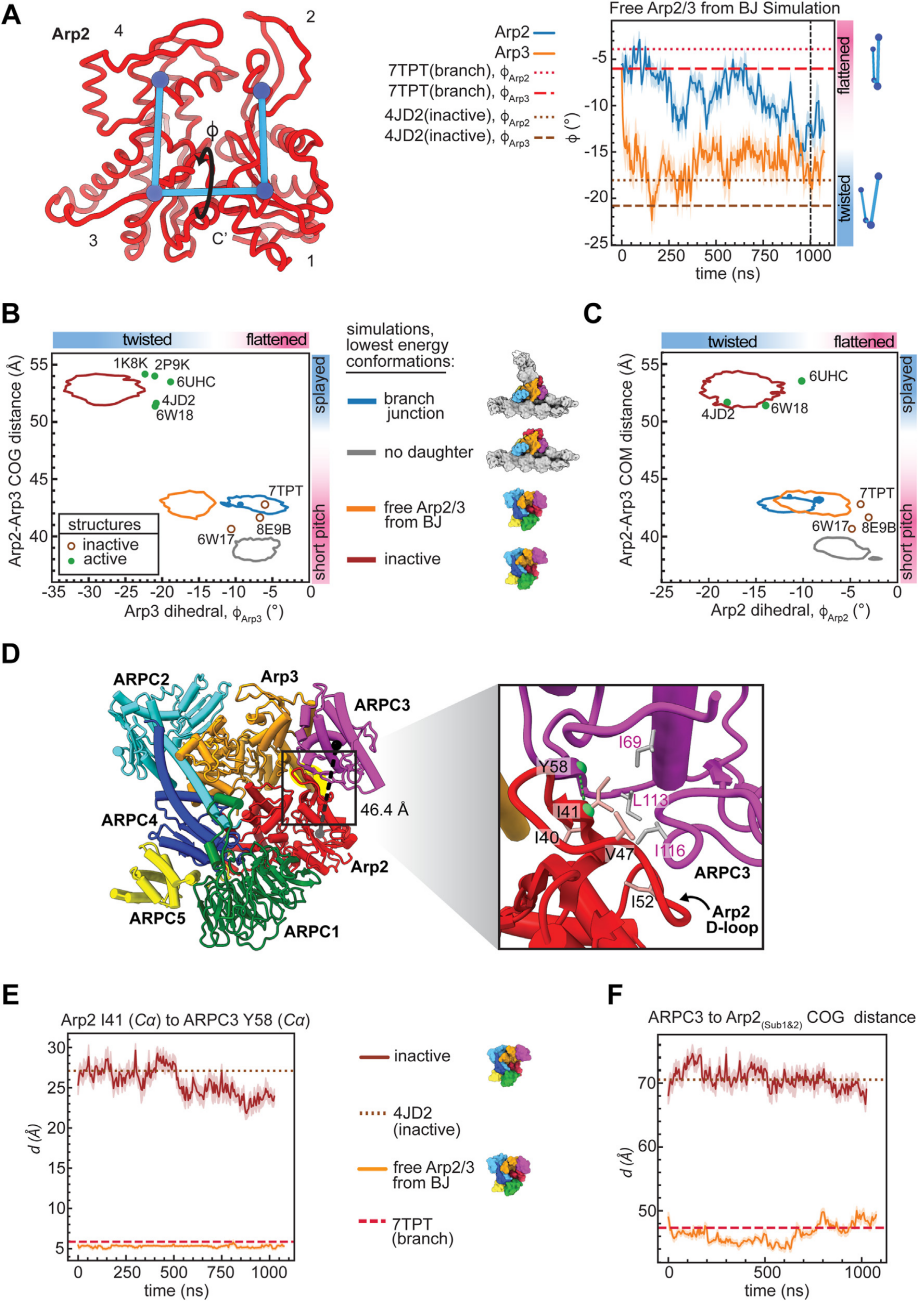
**Unbiased simulations show that flattening and adoption of the short pitch conformation arenot strongly linked.***A*, (*left*) backbone trace of Arp2 showing measurement of the twisting/flattening angle. Subdomains of Arp2 are labeled 1 to 4. (*right*) Plot of Arp2 and Arp3 twisting/flattening angle (φ) *versus* simulation time for the free Arp2/3 complex from branch junction simulation. Arp2 and Arp3 twisting/flattening angles from inactive (4JD2) and the active Arp2/3 complex structure (7TPT) are shown as *dashed* or *dotted lines*, as indicated. *Vertical dashed line* shows the simulation frame used to generate *panel D*. *B*, plot of the Arp3 subunit twisting/flattening dihedral *versus* the Arp2-Arp3 COG, which measures movement into the short pitch conformation. Enclosed regions indicate the most probable conformations in the simulation, as defined by conformations that are within a radius of one free energy unit from the lowest energy conformation. *Circles* show the corresponding measurements for selected active and inactive cryo-EM or X-ray crystal structures. *C*, plot as described in *B*, except the Arp2 twisting/flattening dihedral angle is plotted on the x-axis. *D*, ribbon diagram of the free Arp2/3 complex from branch junction simulation output at 1 μs showing that the Arp2 D-loop maintains contact with ARPC3 even when the complex moves into a short-pitch, twisted conformation. The D-loop of Arp2 is highlighted in *yellow*. The distance between the globular domain of ARPC3 and subdomains 1 and 2 of Arp2 is indicated with a *black line*. Inset shows a zoomed in view of the interaction. *Green dashed line* shows the distance between I41^Arp2^ Cα and Y58^ARPC3^ Cα plotted in *panel E*. *E*, plot showing the distance between Arp2 D-loop and ARPC3 for Arp2/3 complex (active) and (inactive) simulations *versus* simulation time, with 7TPT and 4JD2 plotted for reference. *F*, plot showing the distance between the COGs of Arp2^Sub1&2^ and ARPC3 *versus* simulation time in the Arp2/3 complex active simulation. Arp2/3 complex, actin-related protein 2/3 complex; cryo-EM, cryo-electron microscopy; COG, center of geometry.

A key feature of concerted models is that ligand-induced conformational changes are strongly linked so that the system exhibits high cooperativity and switch-like activation when all ligands are bound (46). In a fully concerted Arp2/3 complex activation mechanism, movement into the short pitch conformation and flattening would be perfectly coupled. In contrast, in the multistep model for activation, these two conformational switches could occur independently and give switch-like activation (14, 24). To investigate potential links between the activating conformational changes, we asked if Arp2 or Arp3 could switch from the flattened to twisted states in the simulations of activated Arp2/3 complex, even though the complex stays in the short pitch conformation (Fig. 2). We used the dihedral angle between the COG of the four subdomains of the Arps as a metric for subunit flattening, as previously described ((16), Fig. 3*A*). During the 1 μs simulation of free Arp2/3 complex from the branch junction, the dihedral angle of Arp3 rapidly moved toward the twisted state and stayed in a short-pitch, twisted conformation for much of the simulation (Fig. 3, *A* and *B*). The short-pitch, twisted conformation was also adopted in Arp2 in the simulation of the branch junction and moderately populated in Arp2 in the simulation of free Arp2/3 complex from the branch junction (Fig. 3*C*). These data indicate that Arp2 and Arp3 can move from a flattened state into or close to the twisted conformation even when they are arranged in the short pitch conformation. This suggests that the two major activating conformational changes in Arp2/3 complex—adoption of the short pitch conformation and subunit flattening—are not tightly linked. Furthermore, the existence of a stable intermediate conformation of Arp2/3 complex, in which one activating conformational change (short pitch adoption) but not the other (flattening) has occurred argues against a concerted mechanism for activation (Fig. 1*A*).

The observation that the Arps undergo subunit twisting when the complex is in the short pitch conformation was unexpected because flattening is thought to increase the buried surface area (BSA) between Arp2 and Arp3 in the short pitch complex (16). However, in addition to contacts between Arp2 and Arp3, other intracomplex interactions appear to stabilize the short pitch conformation, including contacts between the D-loop of Arp2 and a hydrophobic pocket in ARPC3 (Fig. 3*D*). This interaction—which was shown to be important for nucleation activity of the complex (14)—is maintained when the Arps twist (Fig. 3*D* and *E*). In contrast, the Arp2 D-loop/ARPC3 interaction is not observed in structures or in simulations of inactive Arp2/3 complex, in which the two actin-related subunits are in the splayed configuration, because movement into the short pitch conformation moves the centers of mass of Arp2 and ARPC3 apart by ∼20 Å (Fig. 3, *E* and *F*). These data provide an explanation for the apparent stability of the short-pitch, twisted intermediate state; because of the flexibility of the Arp2 D-loop, the ARPC3–Arp2 interaction can stabilize the short pitch conformation regardless of whether the Arps are flattened or twisted. We note that our observations differ from a recent analysis of the activated *S. pombe* Arp2/3 complex which suggested that the twisted conformation is incompatible with the short pitch conformation because of steric clash (see Discussion) (15).

### Contacts with the mother filament stabilize flattened Arp3

**Figure 4:**
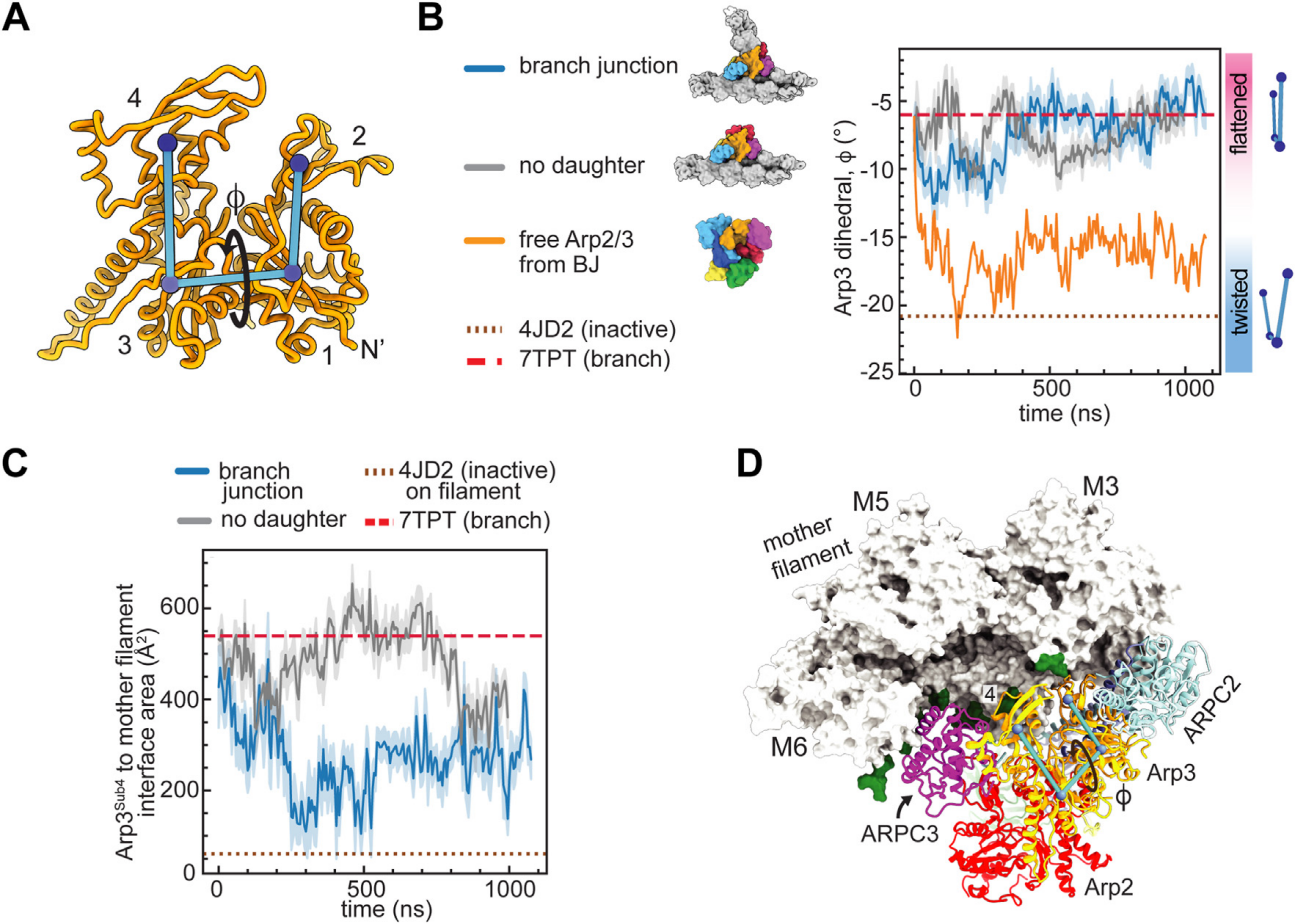
**Contacts with the mother filament stabilize flattened Arp3.***A*, diagram showing the twisting/flattening angle (φ) of Arp3. The four subdomains of Arp3 are labeled 1 to 4. *B*, plot of Arp3 twisting/flattening dihedral angle *versus* simulation time for all unbiased simulations that started in the active conformation. Arp3 twisting/flattening angles from inactive (4JD2) and active Arp2/3 complex structures (7TPT) are shown in *dotted* or *dashed lines*, as indicated. Data for the free Arp2/3 complex from branch junction is replotted from Fig. 3*A*. *C*, plot of the surface area of subdomain 4 of Arp3 buried on the mother filament *versus* simulation time. Buried surface area of subdomain 4 in the branch junction structure (7TPT) and a model of 4JD2 on an actin filament are shown as *dotted* or *dashed lines*. *D*, ribbon and surface representation of the last frame of the branch junction without daughter filament simulation showing residues within actin filament that interact with Arp2/3 complex upon subunit flattening in Arp3. Actin filament residues that interact with ARPC3 or Arp3^Sub4^ in the MF-bound Arp2/3 complex simulation (average contact score > 1, *colored green*) are mapped onto the surface of the actin filament. Subdomain 4 of Arp3 is labeled. The Arp3 dihedral angle that flattens Arp3 is shown as angle φ. Arp2/3 complex, actin-related protein 2/3 complex.

We next analyzed the simulations to determine whether interactions of Arp2/3 complex with the mother filament favor subunit flattening in Arp3, as predicted based on the branch junction structure of *B. taurus* Arp2/3 complex (14). We found that Arp3 stays in or close to the flattened conformation in both the simulation of the branch junction and the simulation of the branch junction without daughter filament (Fig. 4, *A* and *B*). This contrasts the simulation of the free Arp2/3 complex from the branch junction, in which Arp3 adopts twisted states (Figs. 3*A* and 4*B*). The flattened conformation is stabilized by contacts of subdomain 4 of Arp3 with the mother filament, which are maintained throughout the simulations (Fig. 4, *C* and *D*). ARPC3 also interacts with the mother filament during these simulations (Fig. S3). This subunit is bound to subdomains 3 and 4 of Arp3, so flattening of Arp3 brings it into contact with the mother filament. Together, Arp3 subdomain 4 and ARPC3 bury an average of 1600 Å^2^ and 700 Å^2^ with the mother filament in last 750 ns of the simulations of the branch junction and the branch junction without daughter filament, respectively (Fig. S3). These observations support a model in which binding to the mother filament stimulates flattening in the Arp3 subunit. We note that in steered MD simulations in which Arp2/3 complex is pulled from the short pitch to splayed conformation (see below), Arp3 twists but ARPC3 and subdomain 4 maintain contact with the mother filament. In these simulations, clamp subunits ARPC2 and ARPC4 bend to move Arp3 relative to the filament, allowing it to maintain all filament contacts even when twisted (Video S2). Bending of the clamp has not been observed in X-ray crystal structures or cryo-EM structures of the complex, so will be important to determine whether this conformation of the complex is biologically relevant.

### Twisting of Arp2 closes the barbed end groove and weakens its interactions with the daughter filament

**Figure 5:**
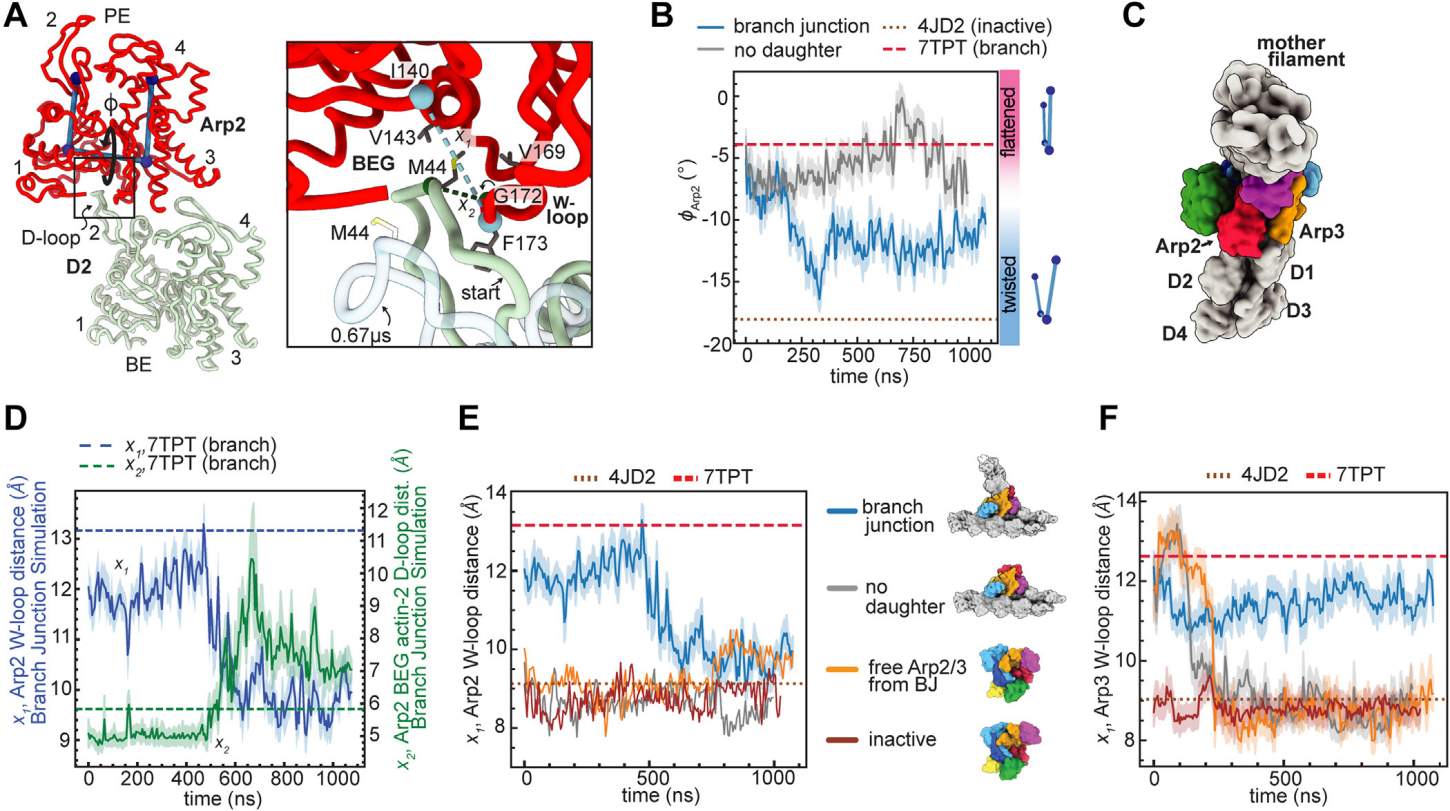
**Twisting of Arp2 closes the barbed end groove and weakens its interactions with the daughter filament.***A*, ribbon diagram showing the interaction of the D-loop of actin D2 with the barbed end groove of Arp2. Arp2 and actin D2 subdomains are labeled 1 to 4. PE: pointed end, BE: barbed end. *Right panel* shows closeup of the interaction with distances measured in B. Actin D2 from 0.67 μs (transparent light blue ribbon) in the branch junction simulation was placed by overlaying Arp2 from the 0.67 μs frame in the trajectory with Arp2 from 7TPT. BEG: Barbed-end groove. Start: position of actin D-loop at the beginning of the simulation. *B*, plot of twisting/flattening angle of Arp2 (φ) in the branch junction and the branch junction without daughter filament (no daughter) simulations. Arp2 twisting/flattening angles from inactive (4JD2) and active Arp2/3 complex structures (7TPT) are shown in *dotted* or *dashed lines*, as indicated. *C*, surface representation of branch junction model (7TPT) showing the interface between Arp2 and Arp3 and the pointed end of the nucleated daughter filament. *D*, plot of the W-loop opening (x_1_) and D-loop to Arp2 distance (x_2_) in the branch junction simulation. See panel A for definition of x_1_ and x_2_. Distances x_1_ and x_2_ in the branch junction structure are plotted for reference. *E*, plot of W-loop distance (x_1_) in Arp2 subunits for all unbiased simulations. Distance x_1_ in the branch junction (7TPT) and inactive Arp2/3 complex structure (4JD2) is plotted as *dashed* or *dotted li*nes, as indicated. *F*, identical to *E*, except x_1_ for Arp3 from each simulation is plotted. Arp2/3 complex, actin-related protein 2/3 complex.

While Arp3 stays near or in a flattened conformation in the simulation of the branch junction, Arp2 moves closer to the twisted state, populating mostly intermediate but sometimes nearly fully twisted states (Fig. 5, *A* and *B*). To better understand how twisting influences the interface of Arp2 with the daughter filament pointed end (Fig. 5*C*), we measured the width of the barbed end groove in the simulation of the branch junction. We found that Arp2 twisting was accompanied by a closure of the Arp2 barbed end groove, as measured by the distance between F173 and I140 in Arp2 (Fig. 5, *A* and *D*). This distance measures curling of the W-loop, a short loop that lines the barbed end groove. When the W-loop curls, it closes a pocket in the side of the barbed end groove. Curling of the W-loop in the simulation of the branch junction was accompanied by ejection of Met44 from the actin D2 D-loop from the pocket (Fig. 5, *A* and *D* and video S3). Met44 has previously been shown to be a target of MICAL enzymes, which oxidizes it to stimulate actin filament disassembly (47). Together, our observations support a model in which flattening/twisting of the Arps allosterically influences the interaction of their barbed ends with actin subunits in the long pitch position. This allostery could play an important role in activation of Arp2/3 complex because it provides a mechanism by which actin filaments could promote nucleation by stimulating subunit flattening.

To further probe the link between twisting/flattening of the Arps and the width of their barbed end grooves, we measured the W-loop position in Arp2 and Arp3 in all four simulations. In simulations lacking the daughter filament, the W-loop of both Arps remained or become curled even if the Arp subunit remained flat, suggesting that insertion of the D-loop of actin into the barbed end groove helps stabilize an open barbed end groove (Fig. 5, *E* and *F*). The W-loop of Arp3 tended to remain uncurled through a greater portion of the simulations than in the Arp2 subunit, suggesting that the open state of the barbed end groove may be more favorable in Arp3 compared to Arp2. This finding may have implications for understanding how WASP-mediated recruitment of actin monomers to each Arp influences the activating conformational changes.

### Splayed Arp2/3 complex maintains approximately the same interface area with the mother filament as short pitch Arp2/3 complex

**Figure 6:**
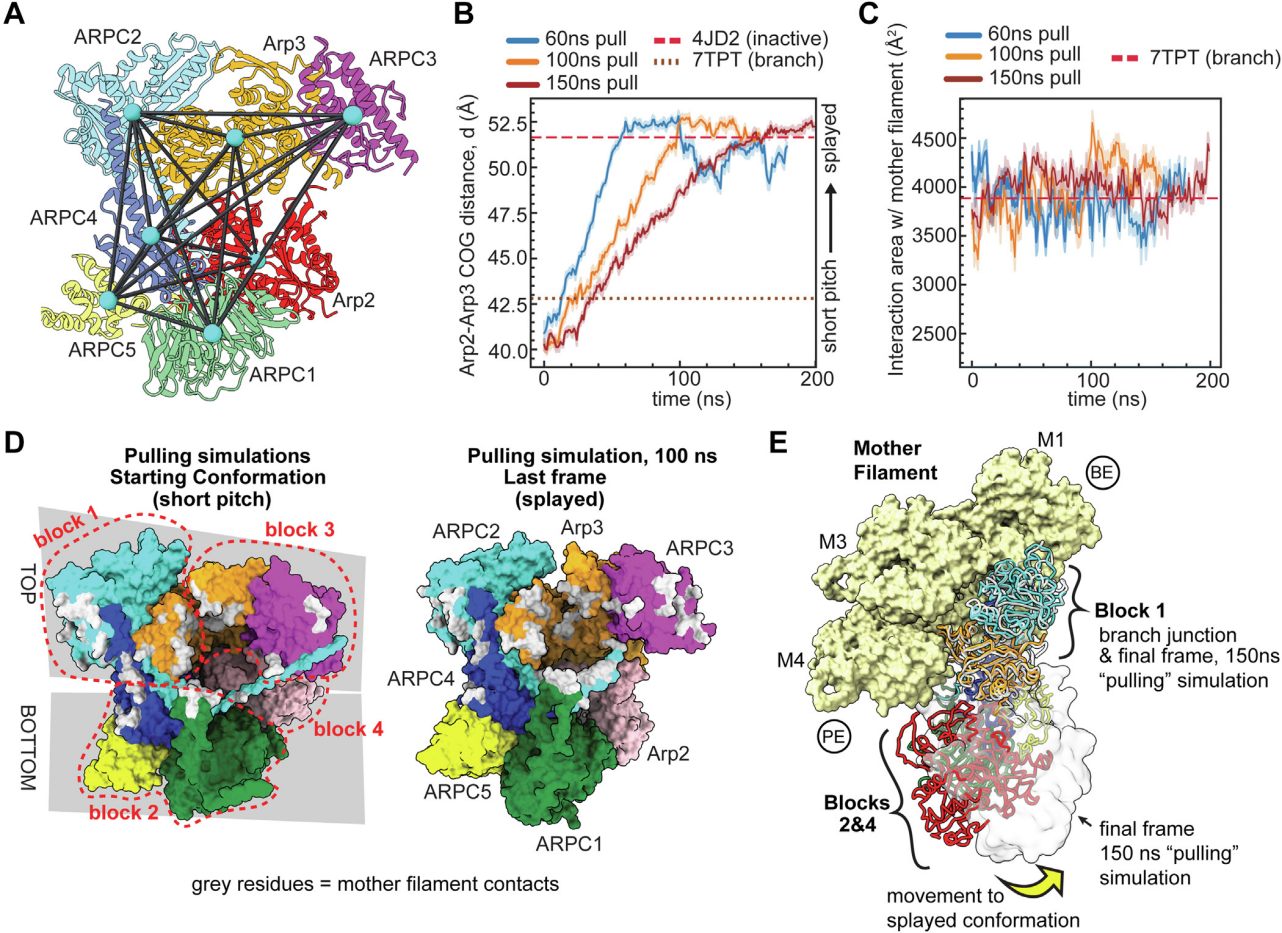
**Splayed Arp2/3 complex maintains approximately the same interface area with the mother filament as short pitch Arp2/3 complex**. *A*, ribbon diagram of inactive Arp2/3 complex (4JD2) showing the center of geometry (COG) of each subunit. For the steered MD simulations, a bias was applied to move the COGs from their positions in the active complex to their positions in the inactive (4JD2) complex. *B*, plot of the distance between the Arp2 and Arp3 subdomains 3 and 4 COGs as a function of simulation time. The same distances for the inactive (4JD2) and branch junction (7TPT) structures are plotted as *dashed* and *dotted lines*, as indicated. *C*, plot of the total interaction area of Arp2/3 complex with the mother filament in all three steered simulations. Data for simulations are shown as an average smoothed over 1 ns (10 frames) for this and all other plots for the steered simulations. The standard deviation over the smoothing window is *shaded*. Interaction surface area in the branch junction (7TPT) is plotted as a *dashed line* for reference. *D*, (*Left panel*) surface representation of Arp2/3 complex rendered using the starting coordinates of the 100 ns pulling simulation. The four rigid blocks that move independently when the complex undergoes subunit flattening (see Video S1) are outlined with *red dashes*. The “*top*” and “*bottom*” rigid blocks that move independently when the clamp twists are indicated with *gray boxes* behind the complex. Residues of Arp2/3 complex that contact the mother filament at the start of the simulation (PyContact calculated contact score >1) are colored *gray*. (*Right panel*) Same as left panel except surface representation is rendered from the final frame of the simulation and residues that have an average contact score > 1 over the last 1 ns of the simulation are colored *gray*. Arp2/3 complex is in the splayed conformation at the end of the simulation. *E*, comparison of mother filament binding contacts of activated (flattened, short pitch) Arp2/3 complex (7TPT) to those of the splayed Arp2/3 complex (final frame of MF-bound 150 ns pulling simulation—rendered in gray ribbon or transparent *gray* surface). The splayed Arp2/3 complex was modeled onto the mother filament by superposing block 1 onto block 1 in the branch junction model. *Yellow arrow* shows movement of blocks 2 and 4 stimulated by clamp twisting. Block 3 is omitted for clarity. BE: Barbed end of mother filament. PE: pointed end of mother filament. Arp2/3 complex, actin-related protein 2/3 complex.

Our MD simulations suggest actin filaments stimulate subunit flattening in Arp3, one of the major activating structural changes in Arp2/3 complex. It is also important to determine whether actin filaments can stimulate the other major activating conformational change: movement into the short pitch conformation. Filaments will stimulate the short pitch arrangement if the short pitch conformation of the complex interacts more favorably with the filament than the splayed conformation. However, there are no structures of Arp2/3 complex in the splayed state bound to the filament, so information about the inactive interface is limited (14, 15). Therefore, to better understand the differences between short pitch and splayed mother filament contacts, we ran SMD simulations using the branch junction without daughter filament system (Fig. 1*B*). We used a harmonic biasing restraint to pull Arp2/3 complex from the short pitch to the splayed conformation while it remained bound to the side of the filament, as described above (Fig. 6*A* and Video S4). The pulling forces were applied over three different time intervals. In all three pulling intervals, the Arp2 and Arp3 subunits move into the splayed arrangement (Fig. 6*B*). The pathway to the splayed conformation is consistent with previous empirical and computational studies; it proceeds *via* a twisting of the clamp subunits (ARPC2 and ARPC4) that rotates the bottom half of Arp2/3 complex—including Arp2, ARPC1, ARPC5 and the globular portion of ARPC4—into the splayed position (14, 15, 36, 38) (Fig. S4, *A–C*). These observations show that the bias applied in the steered MD simulations produces a splayed conformation of filament-bound Arp2/3 complex via a pathway consistent with the available empirical data. We note that the steered simulations change the conformation of Arp2/3 complex in a direction opposite to that occurring during branching nucleation. While it is unclear whether the conformations can interchange on the side of the filament, we expect this set of conformational changes to occur sometime after branch disassembly to reset the complex to the inactive conformation.

To assess how the conformation of Arp2/3 complex influences its contacts with the mother filament, we analyzed the surface area buried with the mother filament during the pulling simulations. Because BSA is roughly correlated to the energy of the binding interface (48), we reasoned that this analysis could provide insight into how binding to actin filaments might influence the short pitch conformational switch.

The total interaction area with the mother filament is relatively constant throughout the simulations, with an average interface area of 3880 Å^2^, 3971 Å^2^, and 3995 Å^2^ during the 60, 100, and 150 ns simulations, compared to 3884 Å^2^ for the branch junction structure (14) (Fig. 6*C*). Furthermore, the residues on Arp2/3 complex that contact the mother filament are nearly identical at the beginning of the simulations, when the complex is in the short pitch conformation, as they are at the end of the simulation, after the complex has been pulled into the splayed conformation (Fig. 6*D* and S4*D*). Examination of the trajectory revealed how contacts to the mother filament are maintained in the splayed conformation. As the clamp twists, the top portion of the complex (Arp3, ARPC3, and the globular domain of ARPC2) stays anchored to the mother filament and maintains the same or similar contacts to those it makes in an activated conformation (Fig. 6*E* and Video S5). The two rigid blocks shown in Fig. 6*E* that make up the bottom of the complex (2 and 4) rotate away from the filament as the clamp twists into the splayed conformation, but these blocks contact the filament almost exclusively through flexible segments that can engage the filament regardless of the conformation of Arp2/3 complex (see below). Therefore, these simulations suggest that clamp rotation and movement of Arp2/3 complex into the splayed conformation may not significantly influence its interface with the mother filament, in agreement with previous modeling experiments (14).

### Flexible segments in Arp2/3 complex maintain conformation-insensitive contacts with the mother filament of actin

**Figure 7:**
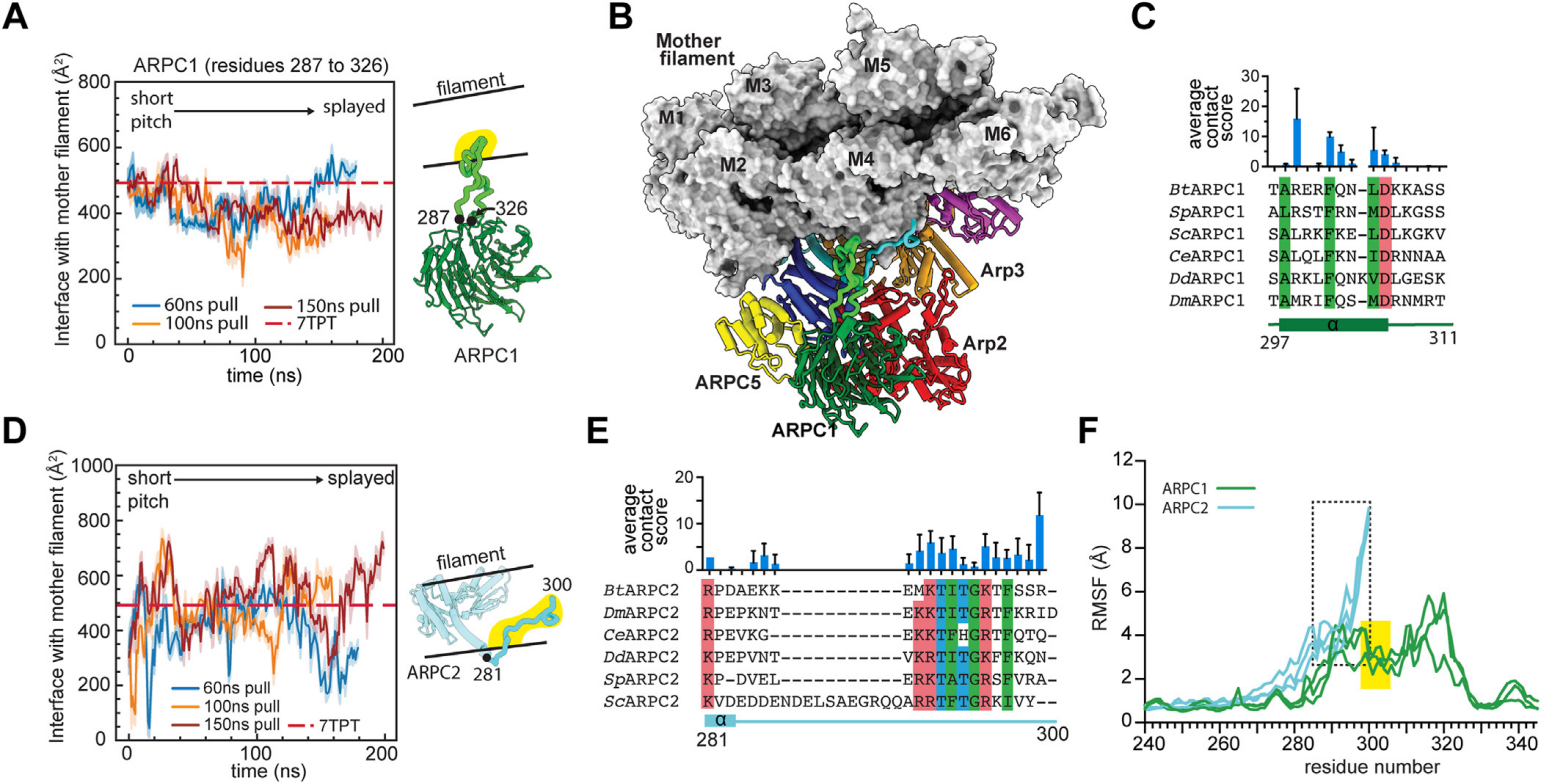
**Flexible segments in Arp2/3 complexmake contacts to the mother filament that are insensitive to the Arp2/3 complex conformation**. *A*, plot of the interaction area of ARPC1 residues 287 to 326 with the mother filament *versus* simulation time for all pulling simulations. *Dashed line* shows the corresponding interaction areas in the branch junction structure 7TPT. *B*, surface and cartoon representation of Arp2/3 complex bound to the mother filament in the last frame of the 100 ns pulling simulation. The two flexible segments from the bottom half of the complex (ARPC1 287–326, *green*, and ARPC2 281–300, *cyan*) are shown in thicker cartoon representation. *C*, sequence alignment of the ARPC1 insert sequence from a diverse range of species showing conserved hydrophobic (*green*) and acidic (*red*) residues. The average contact score over the course of all three pulling simulations is plotted above the sequence for each residue. Error bars: standard deviation. *D*, plot of the interaction area of ARPC2 residues 281 to 300 with the mother filament *versus* the simulation time for all pulling simulations. *E*, sequence alignment of ARPC2 C-terminal extension from a diverse range of species showing conserved hydrophobic (*green*), basic (*red*), or hydrophilic (*cyan*) residues. The average contact score over the course of the three pulling simulations is plotted above the sequence for each residue. Error bars: standard deviation. *F*, plot of the root mean squared fluctuation (RMSF) from the initial conformation for backbone atoms of the ARPC1 insert (*green*) or the ARPC2 C terminus (*cyan*) over the entire simulation, plotted separately for each pulling simulation. *Black dashed rectangle* highlights RMSF values for residues in ARPC2 C-terminal extension with the closest contacts to the mother filament. *Yellow rectangle* highlights RMSF values for residues in the ARPC1 insert with the closest contacts to the mother filament. Arp2/3 complex, actin-related protein 2/3 complex.

In addition to rigid block 1 and 3 in the top of the complex, flexible segments on ARPC1 and ARPC2 from the bottom of Arp2/3 complex remain attached to the mother filament as the complex moves from the short pitch to the splayed conformation. The ARPC1 insert forms a short α helix that binds to a hydrophobic groove in actin filament subunit M4 (13, 14, 15). We showed previously that because the ARPC1 insert is flexible, it can stay bound to the filament throughout the pulling simulation even as the globular portion of ARPC1 moves away from the mother filament (14). The ARPC1 insert helix has several conserved residues and buries an average of 434, 349, and 415 Å^2^ throughout the 60, 100, and 150 ns pulling simulations, respectively (Fig. 7, *A*–*C* and Video S6).

The C-terminal extension in ARPC2 also remains bound to the mother filament throughout most of the simulation. This segment is disordered in all structures except the recent cryo-EM structure of *S. pombe* Arp2/3 complex at the branch junction (15). Recent coarse-grained simulations suggested it may play a role in stabilizing interactions with the mother filament (32). Because it extends from the C terminus of subunit ARPC2—which moves very little during the transition from the splayed to short pitch conformation—the extension stays positioned at the mother filament interface during activation, burying an average of 404, 471, and 525 Å^2^ throughout the entire 60, 100, and 150 ns pulling simulations, respectively. Several residues in the C-terminal extension of ARPC2 are broadly conserved (Fig. 7*E*), suggesting the potential importance of this interaction. However, while the extension maintains contact with the mother filament throughout the trajectory, it remains relatively flexible, binding several different surfaces during the simulation (Fig. 7*F* and Video S6). This observation is consistent with previous structural data showing this segment is either completely disordered or has weak electron density, even when bound to the mother filament (14, 15).

## Discussion

Here we use steered and unbiased all-atom MD simulations to investigate the conformational pathway of WASP-mediated Arp2/3 complex activation. WASP stimulates movement of the Arps into the short pitch conformation (17, 18, 49), but WASP was not included in any of our simulations, which instead focused on the role of actin filaments in activation. Using simulations to investigate the influence of WASP on activating structural changes will be an important next step, especially considering that while some experiments indicate that the primary function of WASP and WASP recruited actin monomers is to stimulate the short pitch conformation (17, 50), other experiments point to an additional WASP-mediated function in the activation process (18). In addition, our studies included only one nucleotide state in the Arps, so it will be important to use simulations to investigate how other nucleotide states influence activating conformational changes. These studies will provide insights into why the nucleotide is required for activation and how it controls the stability of branches (41, 51, 52, 53).

A key conclusion from this work is that the two main activating conformational changes in Arp2/3 complex, movement into the short pitch conformation and subunit flattening, are unlikely to be concerted. Specifically, we show that the complex can adopt twisted states of Arp2 and Arp3 when the Arps are arranged into the short pitch conformation. These observations support a multistep model for activation, in which flattening and movement into the short pitch conformation can occur in separate steps and can be stimulated by different activating factors. An activation pathway that can be separated into multiple distinct steps has important implications for regulating the complex. First, it provides a mechanism to program triggered WASP release into the reaction mechanism. Previous experiments have shown that WASP is released before or concurrently with nucleation (54). This feature of the activation mechanism is thought to prevent unproductive connections between growing actin networks and membrane-bound WASP, which decrease pushing forces of the network against the membrane (54). In the multistep activation pathway, WASP (and actin monomer binding) could stimulate movement into the short pitch conformation, and subunit flattening stimulated by actin filaments could stimulate WASP release.

Another important advantage of a multistep activation model is that it would permit switch-like behavior of Arp2/3 complex at a wide range of concentrations of actin filaments, WASP and WASP-recruited actin monomers (46). In contrast, concerted models of multisignal activation pathways have switch-like behavior only at low concentrations of activators; moderate to high concentrations cause rheostat-like activation, where each activating factor can trigger some activity on its own (46). Therefore, a multistate model would allow for tighter regulation of Arp2/3 complex in cellular contexts where the local concentrations of activated WASP, actin monomers, and preformed actin filaments are high. We note that while our data support a multistep model, it is possible that there is some conformational coupling between the activation steps, so that the mechanism has some aspects of both a multistate and a cooperative model. This coupling could explain the reported cooperativity between WASP and actin filaments in binding to the complex (22, 23).

Our data points to the existence of stable intermediate conformations of Arp2/3 complex during the activation process. Specifically, we show that Arp2 and Arp3 can move into or part way toward the twisted conformation even with the complex in the short pitch state. Because of the limits on computation time, we were not able to assess whether the short pitch twisted states persist over periods longer than a microsecond. We note that during the simulations, Arp2/3 complex never adopted a state in which both Arp2 and Arp3 were fully twisted and short pitch (Fig. 3, *B* and *C*). However, we were able to create a model of a fully short-pitch/twisted conformation by superposing the half of Arp2 and Arp3 bound to the clamp subunits from the active structures with twisted Arp2 and Arp3 with only minimal steric clash, which could be relieved through side chain minimization (Fig. S5). Therefore, we anticipate that with longer simulation times, this fully twisted/short-pitch conformation would be adopted. We note that we cannot eliminate the possibility that the short pitch twisted state we observe here is merely a transiently populated, high-energy state.

In contrast, when we attempted to model a completely splayed/flattened conformation of the complex using the same procedure, we found clashes that could not be relieved by sidechain minimization or backbone remodeling of known flexible regions (Fig. S5). This may indicate that the flattened conformation is not stable when Arp2/3 complex is in the splayed conformation. Therefore, while there is currently no evidence for an obligatory sequence of binding events by activating factors, steric effects may require that Arp2/3 complex moves out of the splayed conformation before Arp2 or Arp3 flatten. Given that flattening of the Arps aligns residues in the nucleotide binding cleft for ATP hydrolysis (14, 16, 19, 55), stimulating this step only after the subunits are already aligned into a filament-like short pitch arrangement may help prevent unproductive hydrolysis of the nucleotide.

Our simulations showed that short pitch Arp2/3 complex is stable for at least a microsecond even without interactions with actin filaments or WASP. This was not surprising given the typical rates of rigid body motions of large groups of atoms (μs-ms) (45). We expect that with longer simulation times, the complex would relax into a splayed conformation. In contrast, movement of the Arps from flattened to twisted conformations occurred within the microsecond simulations. Although this transition was context-dependent (*e.g.*, it did not occur in Arp3 when the complex was filament-bound), it suggests a lower energy barrier separates movement from flat to twisted states than short pitch to splayed conformational states. Because both subunit twisting and movement into the splayed conformation would be expected to favor branch disassembly, this observation may have important implications for understanding how proteins like GMF and Coro7 stimulate branch disassembly upon binding to the complex (56, 57).

Using steered all-atom simulations, we showed that contacts between Arp2/3 complex and mother filaments remain largely unchanged as Arp2/3 complex transitions from the splayed to the short pitch conformation, suggesting contacts with actin filaments do not trigger the short pitch conformational change. These observations are consistent with crosslinking experiments using dual-cysteine engineered *S. cerevisiae* Arp2/3 complex, which showed that actin filaments do not stimulate the short pitch conformation (24). However, these results differ from Förster resonance energy transfer measurements on *S. pombe* Arp2/3 complex, which showed that probes on the C termini of Arp2 and Arp3 have greater Förster resonance energy transfer efficiency when the complex binds actin filaments (58). Empirical methods and the simulations presented here indicate the C termini of the Arps exhibit flexibility that could influence interpretation of these measurements (14, 19, 59). Analysis of the recent cryo-EM structure of *S. pombe* Arp2/3 complex at branch junction suggested that movement into the splayed conformation decreases the interaction interface with the mother filament by ∼35%, indicating an increased binding energy for the activated state that could be used to stabilize the short pitch conformation (15). A key difference in the *S. pombe* Arp2/3 complex analysis is that it was assumed that the ARPC1 insert and the ARPC2 extension—which are disordered in the structure of inactive *S. pombe* Arp2/3 complex (16)—do not contact the mother filament when the complex binds filaments in an inactive state. Our data here suggest that both of these segments make conformation-insensitive contacts that provide over ∼800 Å^2^ of BSA with the mother filament. Nonetheless, interaction surface areas are only roughly correlated with binding energies (48), and small differences in the contacts could cause significant energetic differences. Therefore, additional biochemical/biophysical methods to probe the relationship between the conformation and binding states of Arp2/3 complex will be an important next step.

## Experimental procedures

### Simulation methods

#### Construction

Four systems were created for MD simulation studies. The “branch junction” consists of Arp2/3 complex, the mother filament (10 actin subunits), and daughter filament (4 actin subunits) with initial configurations derived from the Ding *et al*. structure, 7TPT (14). The “branch junction without daughter filament” is the same but only contains Arp2/3 complex and the mother actin subunits. “Free Arp2/3 complex from branch junction” contains only Arp2/3 complex in the active configuration from 7TPT. “Free inactive Arp2/3 complex” complex is constructed from the X-ray crystal structure of Arp2/3 complex in an inactive state and bound to the inhibitor protein GMFγ (4JD2) (38). The nucleotide states for each system are described in the main text. A Mg^2+^ ion was modeled in the nucleotide binding clefts based on its position in the structures. All residues of each actin or Arp2/3 complex subunit were included in the simulations. The following residues were missing from 7TPT: Arp3, 1 to 2, 417 to 418; Arp2, 1 to 3, 389 to 394; ARPC1, 1, 365 to 372; ARPC2, 284 to 300; ARPC3, 1, 175 to 178; ARPC5, 1 to 9, 27 to 34. The following residues were missing from 4JD2: Arp3, 1 to 2, 40 to 51, 356 to 359, 417 to 418; Arp2, 1 to 3, 43 to 50, 389 to 394; ARPC1, 289 to 318; ARPC2, 284 to 300; ARPC3, 1, 36, 102, 121, 175 to 178; ARPC4, 1 to 2; ARPC5, 1 to 8, 29 to 30, 34 to 35; Missing residues were modeled by hand using Coot or PyMol or automatically with Modeller (60, 61). The C terminus of ARPC2 was modeled as an extended β-strand that protrudes into the solvent. These systems were constructed and equilibrated as previously described (14, 62). Briefly, all systems were built using VMD 1.9.3 and parameterized using a CHARMM22+CMAP forcefield with TIP3P water (43). Bound nucleotides and surrounding water were modeled in the nucleotide binding cleft of each actin or Arp2 and Arp3 protein as previously described (63, 64). Each system was solvated in water such that there is at least 1 nm of water in each direction surrounding the protein. Potassium (K+) and Chloride (Cl-) ions were added so that each system was neutralized, and the resulting concentration of salt was 50 mM.

### Equilibration

Systems were equilibrated in several steps using NAMD 2.14 (65).

(1) Minimization: Energy minimization was carried out in four stages, each for 1000 time steps with 10 kcal/mol/Å^2^ restraints on different groups of atoms, as previously described (63). For the first stage, the protein, nucleotide, nucleotide-bound magnesium ions, and nucleotide waters (within 5 Å of the magnesium ion) were restrained. In the second stage, the protein backbone, nucleotide, nucleotide-bound magnesium ions, and nucleotide-proximal waters were restrained. Third, the nucleotide, nucleotide-bound magnesium ions, nucleotide-proximal waters, and finally, only nucleotide-bound magnesium ions and nucleotide-proximal waters were restrained.

(2) Heating/Equilibration: 10 kcal/mol/Å^2^ restraints were applied to the protein backbone, nucleotide, nucleotide-bound magnesium ions, and nucleotide-proximal waters (As in the second stage of minimization). The system was heated from 0 K to 310 K over 100 ps, using a Langevin Thermostat in the NVT ensemble. Immediately after heating, the system was run in the NPT ensemble with a target pressure of 1 atm using the Langevin piston Nose-Hoover method in NAMD 2.14. Equilibration with the same restraints as the heating stage was carried out over five cycles of 200 ps, with each stage reducing the restraint coupling by half. After the first five cycles, an additional equilibration of 200 ps was run with constraints set to 0.1 kcal/mol/Å^2^. A final cycle of equilibration was run for 400 ps without any constraints on any groups of atoms.

### Production

#### Unbiased MD

For the production runs, a python script (psf2itp) created by the CHARMM-GUI developers was used to convert the equilibrated system topologies and coordinates from NAMD format to GROMACS compatible files with the same forcefield and MD parameters as in the equilibration step (66). Each system was relaxed for an additional 10 ns using GROMACS 2018 (67). To prevent rotation of the simulation box; 10 kcal/mol/Å2 constraints were applied to backbone atoms of the four terminal mother actin subunits (chains L, M, T, and U in 7TPT).

#### Biased MD

PLUMED plugin was used to implement steered MD in our simulations (68). The MOVINGRESTRAINT function was used to apply a time-dependent harmonic potential starting from the Arp2/3 complex short-pitch configuration to the splayed configuration for the two systems without daughter actin. The collective variables used for the biasing were the COG distances between each of Arp2/3 complex domains, as defined by their Cα positions. The target values of the collective variables were calculated from the crystal structure of 4JD2 (38). The biasing simulations used a force constant of 10,000 kJ/mol/A^2^ for different pulling durations- 60 ns, 100 ns, and 150 ns. In addition, the pulling simulations were continued by biasing the DRMSD, which is the distance RMSD between the Cα of the domains of each frame in the trajectory to the Cα of the domains of the 4JD2.

#### Analysis

Simulation analysis was performed using the mdtraj library (version 1.9.4) in python (69). Additional contact analysis and scoring was carried out using GUI-based interface PyContact (70). Trajectory and structure files were also visualized in VMD 1.9.3, PyMOL, and ChimeraX (71).

Subdomains (1–4) of Arp2 and Arp3 were defined as shown in Table S1, with only backbone atoms used in the COG calculations. Each of the subdomains were mapped into single coarse-grained beads using the custom mapping script written in python and then used for our calculations (Available on manuscript github https://github.com/hocky-research-group/BranchSimulation2023). The subunit twisting/flattening was measured by computing the dihedral angle defined by the four subdomains (subdomain 2, 1, 3, and 4). Subdomains 3 and 4 were used to calculated the distance between Arp3 and Arp2 so that the simulation data could easily be compared to X-ray crystal structures, in which subdomains 1 and 2 of Arp2 are often partially or completely disordered (11, 38).

To define clamp twisting, we used the centers of geometry of four small sets of backbone atoms that were close to atoms used in Shabaan *et al*. (16) (Table S2). The custom mapping script described above was used to map the all-atom selections into coarse grained atoms. The clamp twisting angle was measured by computing the dihedral angle of Bead 1–Bead 2–Bead 3–Bead 4. For BSA calculations, we excluded noninteracting subunits of the mother actin filament (MA1, MA0, MA5, MA7, and MA8 using nomenclature in Ding *et al.* (14)) in our calculations to avoid high computational cost and memory. The Shrake and Rupley algorithm in the mdtraj library was used for computing the solvent accessible area (SASA) of each residue of the selected group (69, 72). The SASAs were used to compute the approximate BSA using the equation:

BSA=12(SASA1+SASA2−SASA12)

where *SASA*_*1*_ is the *SASA* of the first group (excluding the second), *SASA*_*2*_ is the *SASA* of the second group (excluding the first), and *SASA*_*12*_ is the *SASA* including both groups. Clashes in the models presented in Fig. S4 were calculated in ChimeraX using the default settings, and side chain minimization was carried out in Phenix (71, 73). Root mean squared fluctuation calculations were carried out in VMD (74).

## Data availability

Scripts and input files used for system construction, data collection and analysis, and figure preparation can be found in the Hocky group GitHub repository (https://github.com/hocky-research-group/BranchSimulation2023). Simulation data saved every 100 ns can also be found in the GitHub repository. All other input or data files are available upon request.

## Supporting information

This article contains supporting information (17, 18, 24, 71, 75, 76).

## Conflict of interest

The authors declare that they have no conflicts of interest with the contents of this article.
