# Supplementary material for: Molecular dynamics simulations support a multistep pathway for activation of branched actin filament nucleation by Arp2/3 complex
Source: J Biol Chem. 2023 Aug 16;299(9):105169. doi: 10.1016/j.jbc.2023.105169 (PMC10514467; doi:10.1016/j.jbc.2023.105169)
Supplement: Supporting information [file mmc1.pdf]

Molecular dynamics simulations support a multi-step pathway for activation of branched actin filament nucleation by Arp2/3 complex

Yuvraj Singh[1], Glen M. Hocky[1,2*] and Brad J. Nolen[3*]

1. Department of Chemistry, New York University. 2. Simons Center for Computational Physical Chemistry, New York University. 3. Department of Chemistry and Biochemistry, Institute of Molecular Biology, University of Oregon.

*To whom correspondence should be addressed: hockyg@nyu.edu, bnolen@uoregon.edu

	**Arp3**	**Arp2**
**Subdomain 1**	6-32, 78-153, 375-408	7-33, 74-150, 352-387
**Subdomain 2**	33-37, 60-77	34-38, 55-73
**Subdomain 3**	154-196, 295-344, 362-374	151-185, 277-326, 339-351
**Subdomain 4**	197-282	186-265

Table S1: Definition of subdomains 1-4 in *Bos taurus* Arp3 and Arp2. Backbone atoms in the listed residues were used for center of geometry calculations.

	**Subunit**	**Residues**
**Bead 1**	ARPC4	14-50, 63-73, 117-132
**Bead 2**	ARPC4	3-13, 51-62, 74-77, 135-142
**Bead 3**	ARPC2	125-262
**Bead 4**	Arp3	6-32, 78-153, 375-408, 33-37, 60-77

Table S2: Definition of centers of geometry in *Bos taurus* Arp3, ARPC2 and ARPC4 used for calculating the clamp twist angle. Backbone atoms in the listed residues were used for center of geometry calculations.

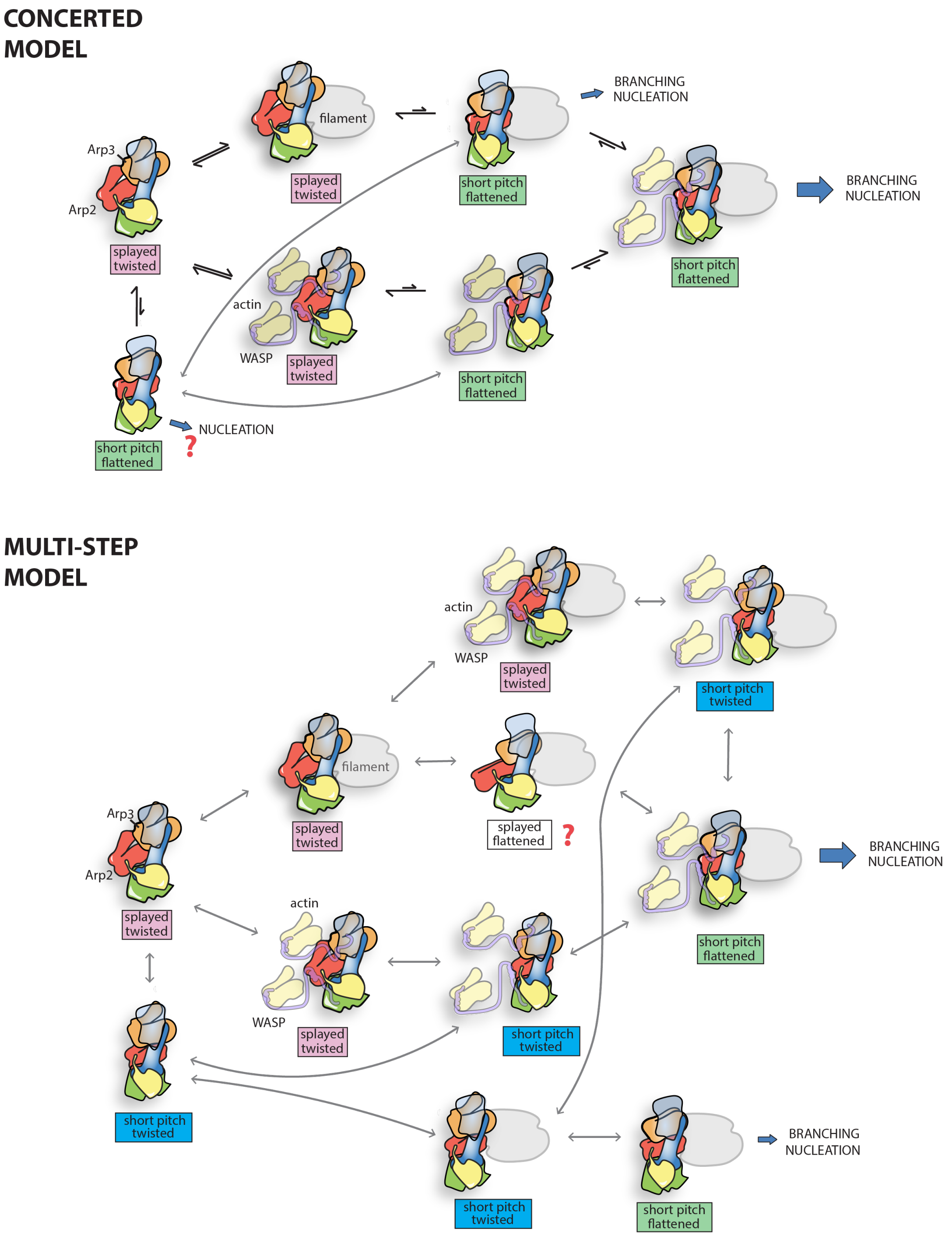

Figure S1: Detailed models for concerted and multi-step pathway for activation of Arp2/3 complex. These schematics show the relationship between conformation and binding state in more detail than in Fig. 1A. Key additions relative to Fig. 1A are: the short pitch conformation can be weakly adopted even in the absence of WASP (1, 2), Arp2/3 complex can adopt the short pitch or splayed conformation when bound to WASP (1, 2), and Arp2/3 complex from some species shows NPF-independent activity (3). It is unknown (red question mark, concerted model) whether the NPF-independent activity of Arp2/3 complex

requires preformed filaments, though filaments activate short-pitch crosslinked *S. cerevisiae* Arp2/3 complex (4). Neither schematic shows individual WASP binding steps. Biochemical data indicate WASP binds more tightly to the Arp2 site (5), and that there may be differences in the contribution of WASP binding at each site to stimulating the short pitch conformation (2). The splayed/flattened state (red question mark, multi-state model) may not be adopted because of steric clash (see discussion).

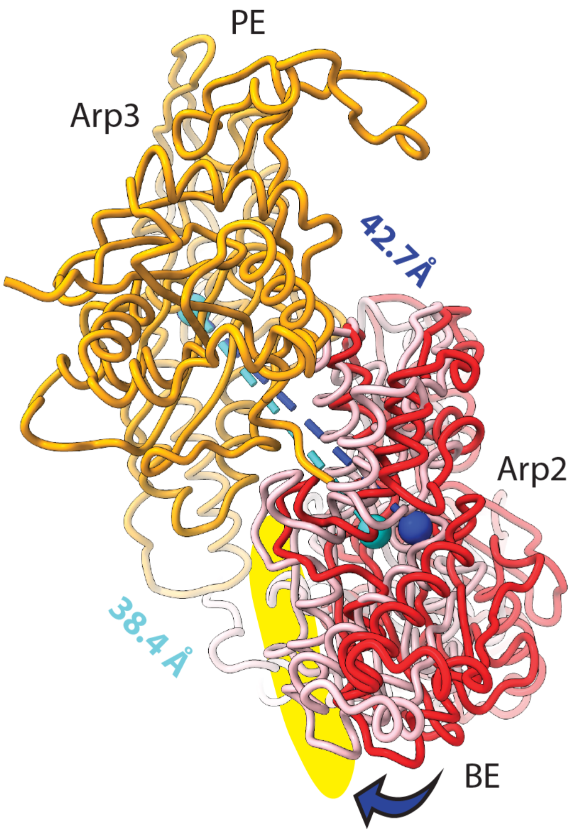

Figure S2: Arp2 in the branch junction without daughter filament simulation tilts into a conformation incompatible with actin D1 binding.

Ribbon representation of Arp3 (orange) and Arp2 (red) from the branch junction structure (7TPT). Arp2 from the MF-bound Arp2/3 complex simulation at 0.76 µs is shown in pink and is positioned by overlaying the Arp3 backbone from the simulation onto Arp3 from the branch junction structure. The centers of geometry of subdomains 3 and 4 of Arp3 and Arp2 from each of the structures are shown as spheres and connected with blue (branch junction) or cyan (simulation) dashed lines. The yellow region shows where actin D1 would clash with Arp2 in the tilted conformation observed in the simulation. PE: pointed end, BE: barbed end.

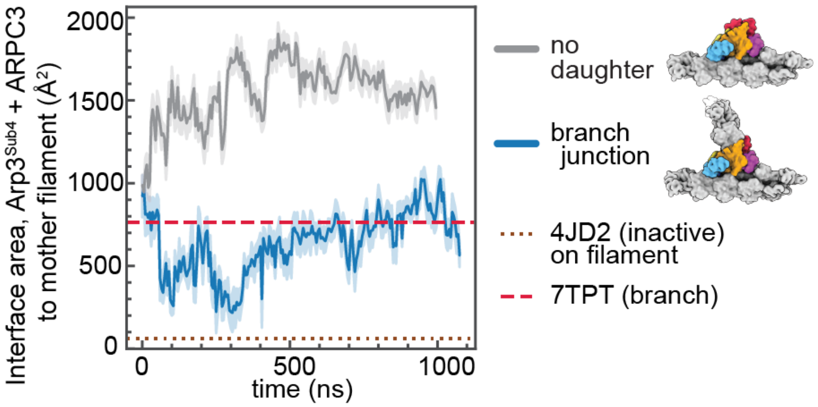

Figure S3: Contacts with the mother filament stabilize flattened Arp3.
Plot of the area of subdomain 4 of Arp3 and ARPC3 buried upon interaction with the mother filament versus simulation time. Buried surface area of the same regions in the branch junction structure (7TPT) and a model of inactive Arp2/3 complex (4JD2) on an actin filament are shown as dashed or dotted lines, respectively.

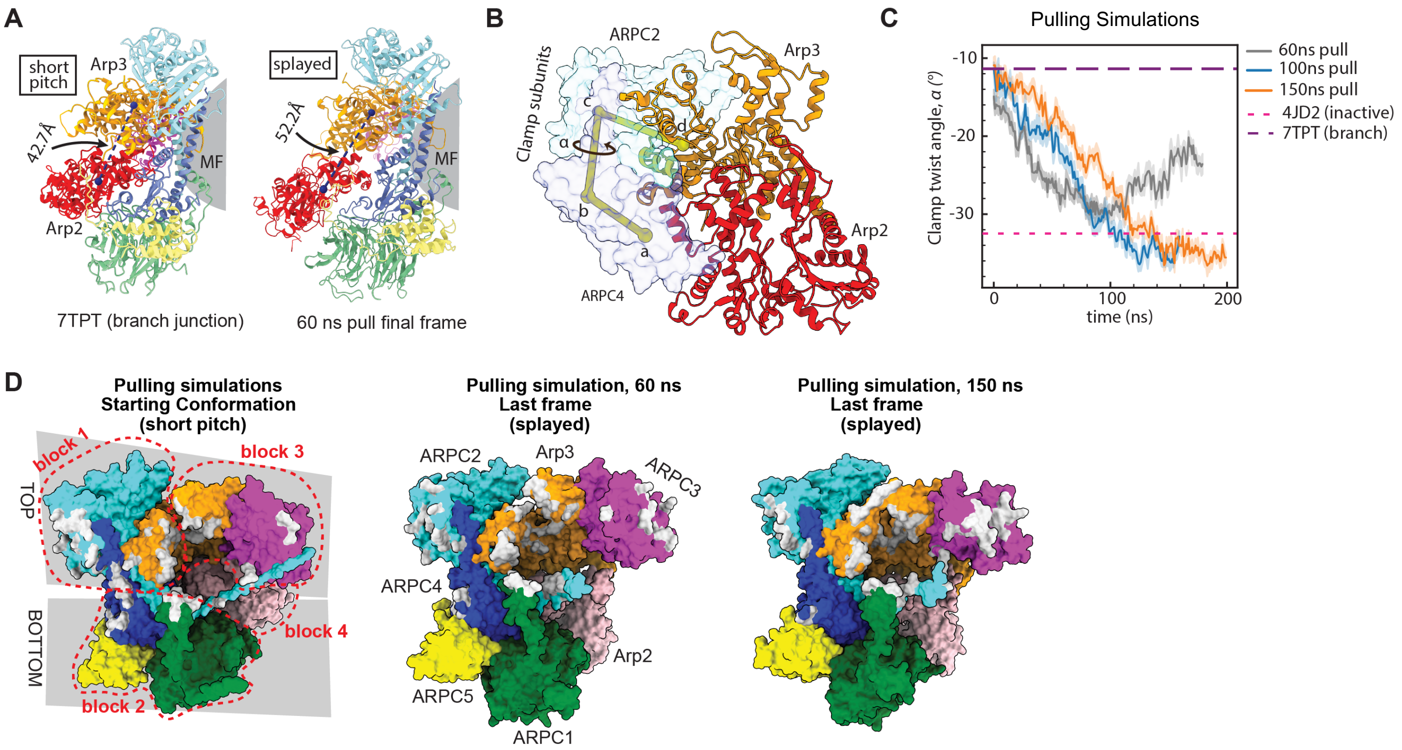

Figure S4: Steered simulations generate a splayed filament-bound Arp2/3 complex structure.
(A) Ribbon diagram of Arp2/3 complex from the branch junction structure and from the 60 ns pulling simulation showing the distances between subdomains 3 and 4 of Arp3 with subdomains 3 and 4 of Arp2. This distance measures movement of the complex into the short pitch position. The approximate position of the mother filament is shown in shaded grey. MF: Mother Filament. **(B)** Ribbon representation of Arp2 and Arp3 from the branch junction structure with the clamp subunits shown as semi-transparent surface. The dihedral angle (α) used to measure clamp twisting is shown in yellow. Residues used to define the centers of geometry marked a, b, c, and d are listed in the Supplementary Materials. **(C)** Plot of the clamp twisting angle (α) versus simulation time for all three pulling simulations. The clamp twisting angle for the branch junction structure (7TPT) and for an inactive Arp2/3 complex structure (4JD2) are shown as dashed lines. **(D)** (Left panel) Surface representation of Arp2/3 complex rendered using the starting coordinates of the MF-bound Arp2/3 complex 100 ns pulling simulation. The four rigid body blocks that move independently when the complex undergoes subunit flattening and clamp twisting (see Video S1) are indicated with red dashes. The "top" and "bottom" rigid body blocks that move independently when the clamp twists are indicated with grey boxes behind the complex. Residues of Arp2/3 complex that contact the mother filament at the start of the simulation (PyContact calculated contact score >1) are colored grey. (Right panel) Same as left panel except surface representation is rendered from the final frame of the simulations and residues that have an average contact score > 1 over the last 1 ns of the simulations are colored grey. Note that the complex is in the splayed conformation at the end of the simulations.

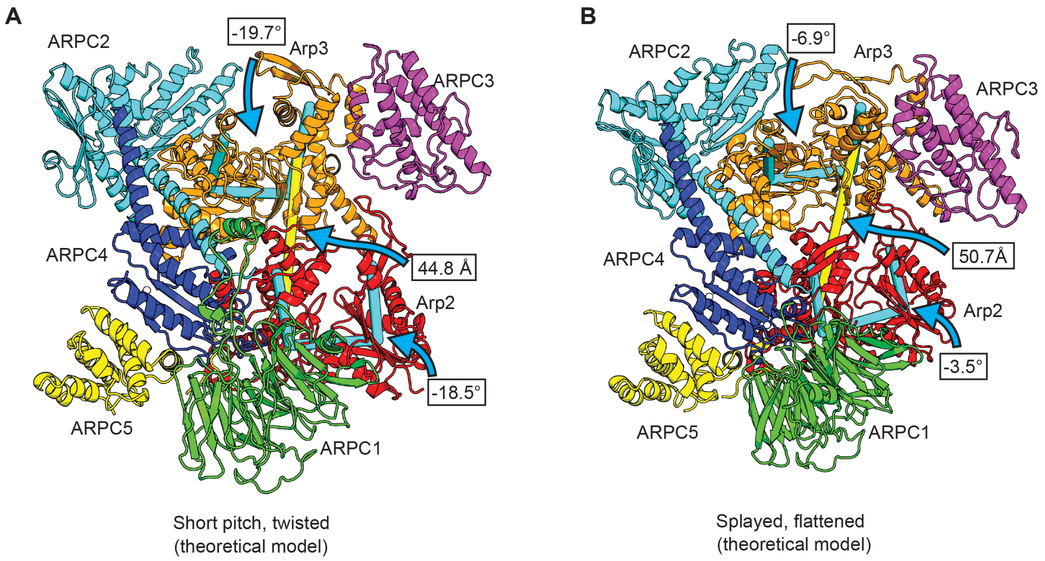

C

structure	# of clashes
7TPT (branch)	13
4JD2 (inactive)	5
short pitch, twisted (side chain min.)	8
splayed, flattened (side chain min.)	53

Figure S5: The short pitch and fully twisted conformation of Arp2/3 complex can be modeled without significant clash.

(A) Cartoon representation of Arp2/3 complex modeled into the short pitch, twisted conformation. Arp3 was constructed by superposing the backbone atoms of (well-ordered) residues in subdomains 1 and 2 of Arp3 from the inactive structure (4JD2, Arp3:6-32,78-153,375-408,33-37,60-77) with the same atoms in the branch junction structure (7TPT). Subdomains 3 and 4 from the superposed inactive structure along with ARPC3 were then used to replace the corresponding residues in the branch junction structure. Arp2 was constructed by superposing the backbone atoms of well-ordered residues in subdomains 3 and 4 of Arp2 in the inactive structure (4JD2, Arp2: 186-265, 151-185, 266-326,339-351) with the same atoms in the branch junction structure (7TPT). Subdomains 1 and 2 from the superposed inactive structure were then used to replace the corresponding residues in the branch junction structure. The dihedral angles that measure subunit twisting/flattening in the Arps are shown in cyan. The distance between the center of geometry of subdomains 3 and 4 of Arp3 and subdomains 3 and 4 of Arp2 is shown in yellow. **(B)** Cartoon representation of Arp2/3 complex modeled into the splayed and flattened conformation. Arp3 in this model was constructed by superposing the backbone atoms of (well-ordered) residues in subdomains 1 and 2 of Arp3 from the branch junction structure with the same atoms in the inactive structure (4JD2). Arp2 in this model was constructed by superposing the backbone atoms of well-ordered residues in subdomains 3 and 4 of Arp2 with the same atoms in the branch junction structure (7TPT). **(C)** Table showing the number of clashes in the experimentally determined versus modeled structures. Clashes were identified with the clash command in ChimeraX (6).

Captions for supplemental videos

Video S1: Activation of Arp2/3 complex requires two major sets of conformational changes. Morph of Arp2/3 complex using the inactive Arp2/3 complex crystal structure 4JD2 as a starting point and the branch junction structure of Arp2/3 complex (7TPT) as the end point. The two major types of activating conformational changes can be measured by dihedral angles (defined by centers of geometry of groups of backbone atoms in the complex – see methods). Clamp twisting is shown with a yellow dihedral, whereas subunit flattening is shown with the grey dihedral (Arp2) or the cyan dihedral (Arp3). Note that only residues present in both the structures are modeled in the morph. Arp3, orange; Arp2, red; ARPC1, green; ARPC2, cyan; ARPC3, magenta; ARPC4, blue; ARPC5, yellow.

Video S2: In steered simulations the clamp bends into a conformation not observed in experimentally determined structures. Video shows two views of the 60 ns pulling simulation overlaid onto the globular portion of ARPC2 in the branch junction structure. Arp3 from the branch junction structure is shown in orange. The Arp3 flattening/twisting angle (ϕ_{Arp3}) for the branch junction structure (7TPT), the inactive structure (4JD2), and the simulation are shown in red, yellow, and cyan, respectively. The ARPC4 subunit is shown for the branch junction structure (red), the inactive structure (yellow) and the simulation (cyan). Bending of the clamp repositions Arp3 so that it can retain contacts with the mother filament despite being in the twisted conformation.

Video S3: Twisting of Arp2 closes the barbed end groove and weakens its interactions with the daughter filament. Ribbon diagram of the branch junction trajectory showing the Arp2 subunit (red) and the actin D2 subunit (grey). The x_1 distance (cyan) measures the uncurling of the W-loop. The x_2 distance (green) measures the distance between M44 Cα in actin D2 and G172 C_α in the Arp2 W-loop. The x_2 distance increases after the sidechain of Met44 is ejected from the pocket in the side of the barbed end groove.

Video S4: A steered simulation with 60 ns of pulling generates a splayed filament-bound Arp2/3 complex structure. Video shows the 60 ns pulling simulation from start to finish. Frames were taken at approximately every 13 ns of the trajectory to make this video. The distance between the COG of subdomains 3 and 4 of Arp3 and subdomains 3 and 4 of Arp2 (grey spheres) is shown as a yellow dashed line. Arp3, orange; Arp2, red; ARPC1, green; ARPC2, cyan; ARPC3, magenta; ARPC4, blue; ARPC5, yellow; actin, gray.

Video S5: Splayed Arp2/3 complex stays anchored to the mother filament and maintains contacts it makes in an activated conformation. Ribbon representation of the 150 ns pulling simulation from start to finish. Frames were output every ~7.5 ns for this video. The distance between subdomains 3 and 4 of Arp3 and subdomains 3 and 4 of Arp2 is shown in white. The clamp twisting dihedral angle (α) is shown in red. Arp3, orange; Arp2, red; ARPC1, green; ARPC2, cyan; ARPC3, magenta; ARPC4, blue; ARPC5, yellow; actin, gray

Video S6: Flexible segments in splayed Arp2/3 complex maintain contacts with the mother filament. Video of ribbon representation of the 150 ns pulling simulation from start to finish. Frames were output every ~7.5 ns for this video. The distance between subdomains 3 and 4 of Arp3 and subdomains 3 and 4 of Arp2 is shown with a green dashed line. The distance between the C_α atom of Ile345 (yellow) on the surface of the mother filament (subunit M4) and the center of geometry of the globular domain of ARPC1 and shown with a magenta dashed line. The distance between the ARPC1 helix and the surface of the

mother filament (ARPC1 Phe302 C_α to actin subunit M4 Ile345 C_α) is shown with a yellow dashed line. The ARPC1 insert and the ARPC2 C-terminal extension are shown in thick cartoon representation.

References for supporting information

1. Rodnick-Smith, M., Luan, Q., Liu, S.-L., and Nolen, B. J. (2016) Role and structural mechanism of WASP-triggered conformational changes in branched actin filament nucleation by Arp2/3 complex. *Proc. Natl. Acad. Sci. U.S.A.* **113**, E3834-3843
2. Zimmet, A., Van Eeuwen, T., Boczkowska, M., Rebowski, G., Murakami, K., and Dominguez, R. (2020) Cryo-EM structure of NPF-bound human Arp2/3 complex and activation mechanism. *Sci Adv*. **6**, eaaz7651
3. Rodal, A. A., Manning, A. L., Goode, B. L., and Drubin, D. G. (2003) Negative Regulation of Yeast WASp by Two SH3 Domain-Containing Proteins. *Current Biology*. **13**, 1000–1008
4. Narvaez-Ortiz, H. Y., and Nolen, B. J. (2022) Unconcerted conformational changes in Arp2/3 complex integrate multiple activating signals to assemble functional actin networks. *Curr Biol*. 10.1016/j.cub.2022.01.004
5. Boczkowska, M., Rebowski, G., Kast, D. J., and Dominguez, R. (2014) Structural analysis of the transitional state of Arp2/3 complex activation by two actin-bound WCAs. *Nat Commun*. **5**, 3308
6. Pettersen, E. F., Goddard, T. D., Huang, C. C., Meng, E. C., Couch, G. S., Croll, T. I., Morris, J. H., and Ferrin, T. E. (2021) UCSF ChimeraX: Structure visualization for researchers, educators, and developers. *Protein Sci*. **30**, 70–82
